# *Incarvillea compacta* Maxim ameliorates inflammatory response *via* inhibiting PI3K/AKT pathway and NLRP3 activation

**DOI:** 10.3389/fphar.2022.1058012

**Published:** 2022-10-28

**Authors:** Ji Zhang, Yujing Feng, Shengqiang Han, Xueting Guan, Ziliang He, Chao Song, Lingyun Lv, Qiaoyu Luo

**Affiliations:** ^1^ School of Life Sciences, Huaiyin Normal University, Huaian, China; ^2^ Qinghai Provincial Key Laboratory of Medicinal Plant and Animal Resources of Qinghai-Tibet Plateau, Qinghai Normal University, Xining, China; ^3^ Department of Anesthesiology, Punan Hospital, Shanghai, China; ^4^ Department of Otorhinolaryngology-Head and Neck Surgery, The Affiliated Huaian No. 1 People’s Hospital of Nanjing Medical University, Huai’an, China

**Keywords:** *Incarvillea compacta* Maxim, anti-inflammatory, RNA sequencing, PI3K/Akt signaling pathway, acute gastritis

## Abstract

*Incarvillea compacta* Maxim is a traditional Tibetan medicine used to treat inflammation-related diseases, such as pneumonia, fever, jaundice, and otitis media. However, no studies have examined its anti-inflammatory mechanism. To validate the anti-inflammatory activity of *I*. *compacta* extract (ICE) and its protective effect on acute alcoholic gastritis, Phytochemicals of *I. compacta* were identified using Ultra-performance liquid chromatography quadrupole time-of-flight mass spectrometry (UPLC-QTOF-MS). Lipopolysaccharide (LPS)-induced RAW 264.7 macrophages were used *in vitro* along with an *in vivo* a mouse acute gastritis model. Pro-inflammatory mediators and cytokines were measured using the Griess reagent and Cytometric bead array (CBA) assay. Furthermore, inflammation-related molecules were analysed by Western blotting, RNA-Seq, and real-time quantitative PCR (RT-qPCR). The experimental results revealed that ICE decreased the nitric oxide (NO), IL-6, MCP-1, and TNF-α levels in LPS-stimulated RAW 264.7 cells, and downregulated the expression and phosphorylation of PDK1, AKT, and GSK3β. Moreover, ICE also downregulated the activation of NLRP3. The RNA-Seq analysis revealed that 340 differentially expressed genes (DEGs) response to ICE treatment was enriched in several inflammation-related biological processes. The results of the *in vivo* mouse acute gastritis model showed that ICE significantly reduced inflammatory lesions in the gastric mucosa and remarkably downregulated the expression of iNOS, TNF-α, IL-1β, and IL-6 mRNA in gastric tissue. Therefore, the results of this study obtained scientific evidence supporting the use of *I*. *compacta*.

## Introduction

Inflammation is a defensive response that protects the body against infection and injury. The inflammatory response can be localised to eliminate the injurious agent and remove damaged tissues to restore health (Chen et al., 2018). Infectious and non-infectious factors that cause injury can result in inflammation (Molteni et al., 2016). In acute inflammation, the inflammatory response may last only for a few days, although it can last much longer and develop into chronic inflammation (Varela et al., 2018). Inflammation is not always beneficial, it often causes discomfort, such as pain or itching, and in some cases can even cause severe disease, including hypersensitivity and autoimmune disease (Straub and Schradin, 2016; Montero-Melendez, 2018).

Many cytokines are involved in the inflammatory process that begins when macrophages are triggered by bacterial Lipopolysaccharide (LPS) (Abdulkhaleq et al., 2018). When cytokines bind to their receptors in signal transduction pathways, they alter the receptor conformation, resulting in the phosphorylation of receptors or receptor-related kinases, and then activate a variety of phosphorylation transcription factors (Hughes and Nibbs, 2018). The intracellular signal transduction cascade pathway is also activated. This alters the amounts of substances involved in mediation of the inflammatory response. Intracellular signal transduction allows cells to respond to external stimuli *via* cell membrane or intracellular receptors, and triggers specific biological effects (Romani et al., 2021). Toll-like receptors (TLRs) are important transmembrane proteins in mammals that transmit extracellular antigen recognition information to cells and trigger inflammatory responses (Sameer and Nissar, 2021). TLRs are the main factors mediating immune and inflammatory responses. TLR4 is activated by agonists and initiates downstream signal transduction to prompt a series of reactions *via* MyD88-dependent and -independent TRIF pathways (Yanagibashi et al., 2015). The LPS receptor is a protein complex composed of LPS-binding protein, CD14, TLR, and MD-2. These receptors can form a high-affinity complex, tightly bind with LPS, activate cells, and release proinflammatory cytokines, growth factors, and enzymes (Latz et al., 2003). LPS mainly activates NF-κB, TBK1-IRF3, MAPKs (including ERK, JNK and p38), JAK-STAT1, and various other signal transduction pathways to trigger the expression of inducible nitric oxide synthase (iNOS) (Pålsson-McDermott and O’Neill, 2004). Therefore, identifying natural products that inhibit nitric oxide (NO) production has become a major goal to allow the development of anti-inflammatory agents.

*Incarvillea compacta* Maxim is a perennial herb in the family Bignoniaceae that mainly occurs in Gansu, Qinghai, Yunnan, and Tibet of China (Zhao et al., 2017). As a traditional Tibetan medicine, it is widely used to treat jaundice, stomach ache, and otitis media (Zhang et al., 2016). Two of the earliest Tibetan books, the “Tibetan Medicine Record” and “Jing Zhu Ben Cao”, mentioned the ethnopharmacological uses of this plant (Pengcuo, 1986; Yang, 1991). Among the many pharmacological activities of *I*. *compacta*, its anti-inflammatory effects have attracted much attention, although the underlying mechanism is unclear. Zhao et al. investigated the chemical constituents of *I*. *compacta*, identifying 23 compounds, including phenylpropanoid glycosides, flavonoids, iridoid glycosides, triterpenes, and steroids, among others (Zhao et al., 2017). Wang et al. isolated and identified 10 compounds from the ethyl acetate fraction of a 95% ethanol extract of *I*. *compacta*: methyl linoleate, tricin, trihydroxy-7-megastigmen-9-one, syringaresinol, salcolin A, rhamnazin, dibutyl-phthalate, trimethyl ellagic acid, kaempferol rhamnopyranoside, and tricin glucopyranoside (Wang et al., 2019). In our laboratory, 12 phenylethanoid glycosides were isolated from the roots of *I*. *compacta*, among which Z-3‴-O-methylisocrenatoside and 7-O-metylleucoseceptoside were novel (Shen et al., 2015; Wu et al., 2016).

This work examined the anti-inflammatory effects of an *I*. *compacta* extract (ICE) on RAW264.7 cells and in a mouse acute gastritis model. The differentially expressed genes (DEGs) of RAW264.7 cells in response to ICE treatment were also analysed at the transcriptome scale using RNA-Seq. The results improve our understanding of the mechanisms underlying the anti-inflammatory activity of ICE.

## Materials and methods

### Plant material and reagents

*I. compacta* maxim were purchased from Xining medicinal materials market (Qinghai China) and authenticated by Professor Haifeng Wu, Institute of Medicinal Plant Development, Chinese Academy of Medical Sciences. Voucher specimens (ICM-2019) was deposited at School of Life Sciences, Huaiyin Normal University. 3-(4,5-Dimethylthiazol-2-yl)-2,5-diphenyltetrazolium bromide (MTT) was purchased from BioFroxx (Einhausen, Germany). LPS was obtained from Sigma (St. Louis, MO, United States). Roswell Park Memorial Institute (RPMI) 1,640 was bought from Invitrogen-Gibco (Beijing China). Fetal bovine serum (FBS) was purchased from Corning (New Zealand). Penicillin/streptomycin was obtained from Invitrogen-Gibco (Carlsbad, CA, United States). L-NG-monomethyl-arginine (L-NMMA) was obtained from Beyotime Biotechnology (Nantong, China). Cytometric bead array (CBA) kit was purchased from BD Biosciences (San Diego, CA, United States). Trizol reagent was obtained from Ambion (Waltham, MA, United States). Rabbit monoclonal antibodies against p-PDK1, PDK1, p-AKT, AKT, p-GSK3β, GSK3β and NLRP3 were obtained from Cell Signaling Technology (Danvers, MA, United States). The goat anti-rabbit IgG H&L (HRP) and *β*-actin were purchased from Abcam (Cambridge, UK).

### Preparation of extract and chromatographic analysis

The dried plant powder (50 g) was extracted three times with 70% ethanol (500 ml) at 90°C for 2 h. The crude extract (ICE) was obtained by removal of the solvent under a rotary evaporator (RV 10, IKA, Germany). The phytochemical profile of ICE was carried out using a Waters Acquity UPLC BEH C18 column (2.1 mm × 100 mm, 1.7 µm). The mobile phase consists of 0.1% formic acid (A) and acetonitrile (B) with a linear gradient elution as follows: 2–40% B from 0 to 5 min; 40–100% B from 5 to 15 min. The flow rate of mobile phase was 0.3 ml/min and detection wavelength was set at 260 nm. The sample inject volume was 2.0 μl. The Ultra-performance liquid chromatography quadrupole time-of-flight mass spectrometry (UPLC-QTOF-MS) included a SYNAPT G2-Si HRMS instrument (Waters, Milford, MA, United States) equipped with an electrospray ionization (ESI) interface. The data were collected in both positive and negative ion modes and the full scan range was set between m/z 50 and 1,500. The source temperature was 120°C, and the desolvation gas temperature was 350°C. The cone and solvent removal gas was nitrogen gas and the flow rates of cone and desolvation gas were set at 50 and 600 L/h, respectively. The capillary voltage was set at 2500 V, while the cone voltage was set at 50 V. Leu-enkephalin was used as a reference mass. All data collected were acquired using MassLynx 4.2 software.

### Cell line and culture condition

The RAW264.7 cells were obtained from American Type Culture Collection (Manassas, VA, United States) and maintained in RPMI 1640 medium supplemented with 10% FBS and 1% antibiotics solution at 37°C under 5% CO_2_.

### Determination of NO, TNF-α, IL-6, and MCP-1 production

In briefly, RAW264.7 cells were seeded on 96 well plates at a density of 1 × 10^5^/well and incubated overnight. Cells were pre-treated with different concentration of ICE (0, 12.5, 25, 50, 100 μg/ml) or 100 μg/ml of L-NMMA for 30 min and stimulated by LPS for 24 h. NO content was measured using Griess reagent (Li F. et al., 2019). Cytokines of TNF-α, IL-6, and MCP-1 were detected by CBA according to the manufactures protocol.

### Transcriptome sequencing and bioinformatic analyses

RAW264.7 cells were seeded on 6-well plates at a density of 5×10^6^/well and incubated overnight. Cells were pre-treated with different concentration of ICE for 30 min and stimulated by LPS for 24 h. RNA were extracted with Trizol reagent kit according to the manufacture’s protocol, and were sequenced on a HiSeq 2,500 sequencing platform (Illumina, San Diego, CA, United States). The raw sequencing data were filtered by remove the raw reads that containing adapters or low quality bases, then the obtained clean reads were further mapped and assembled. Expression abundance and variations of each transcription region were quantified by calculate the Fragment per kilobase of transcript per million mapped reads (FPKM) value.

DEGs were defined based on the mRNAs differential expression between control and ICE treated groups. False Discovery Rate (FDR) ≤ 0.05 and absolute fold change ≥2 were the significance threshold for DEGs selecting. To further understand the biological functions of selected DEGs, bioinformatic analyses were performed on Gene Ontology (GO) and Kyoto Encyclopedia of Genes and Genomes (KEGG) databases. Significantly enriched GO terms and KEGG pathways were defined when the calculated *p*-value gone through FDR correction (FDR ≤0.05).

The RNA sequencing data were validated by real time quantitative RT-PCR analyses using Luna universal qPCR kit according to the manufactures protocol on a CFX-96™ Real-Time instrument (Bio-Rad, Hercules, CA, United States). 16 DEGs were selected for real-time quantitative PCR (RT-qPCR) analysis with the GAPDH gene as a house keeping gene to normalize the expression levels of test genes. Primers used in this work were synthesized by Sangon Biotech (Shanghai, China) and were listed in Table 1. The relative gene expression levels were calculated using 2^−ΔΔCT^ method. All of the samples were triplicated.

**TABLE 1:** Primers using for real time quantitative RT-PCR.

Gene symbol	Forward primer 5′-3′	Reverse primer 5′-3′
Csf2	CGG​CCT​TGG​AAG​CAT​GTA​GA	GGG​GGC​AGT​ATG​TCT​GGT​AG
Il1a	ACG​TCA​AGC​AAC​GGG​AAG​AT	TAG​AGT​CGT​CTC​CTC​CCG​AC
Il1b	AGC​TTC​AGG​CAG​GCA​GTA​TC	TCA​TAT​GGG​TCC​GAC​AGC​AC
Il12b	GGC​TCT​GGA​AAG​ACC​CTG​AC	CGT​GAA​CCG​TCC​GGA​GTA​AT
Il18	AAA​GTG​CCA​GTG​AAC​CCC​AG	TGG​CAA​GCA​AGA​AAG​TGT​CCT​T
Il23a	CAG​CTC​TCT​CGG​AAT​CTC​TGC	AGG​GAG​GTG​TGA​AGT​TGC​TC
Lif	CAA​CTG​GCA​CAG​CTC​AAT​GG	CTT​GTT​GCA​CAG​ACG​GCA​AA
Mmp13	AAG​CCT​TCA​AGG​TCT​GGT​CTG	TGG​CTT​TTG​CCA​GTG​TAG​GT
Src	CTG​AGG​CAC​GAG​AAA​CTG​GT	AGG​ATA​TTG​GCG​GCT​CGA​AG
Tlr8	TCT​ATT​TGG​GCT​GGA​ACT​GCT	AAT​CGT​CCT​GAG​GGA​AGT​GC
Tnfsf15	TGT​CAT​TTC​CCA​TCC​TCG​CA	GAT​GTG​GTC​CCT​CGG​AAT​GT
Cd300a	GAT​AGG​CAT​CCA​GAG​CTG​TCC	CCT​GCC​TCT​GGG​AGT​TGA​AT
Hdac7	TTT​GGG​TAC​ATG​ACG​CAG​CA	GGA​TGC​CCA​CAG​AAA​GGG​AT
Klf10	GAC​TTC​GAA​CCC​TCC​CAA​GG	AGT​CAT​ATA​GTG​CAG​CGC​CC
Marco	AGC​AAC​TCC​GTC​AGC​AGT​TC	ATG​CCC​ATG​TCC​CCT​TTG​TT
S1pr1	TGT​TTG​TGG​CTC​TCT​CTG​CAT	GCT​TCG​AGT​CCT​GAC​CAA​GG
Gapdh	CCA​TCT​TCC​AGG​AGC​GAG​AC	GGT​CAT​GAG​CCC​TTC​CAC​AA

The data generated in this study was uploaded to the China National GeneBank DataBase (CNGBdb) with accession number CNP0003554.

### Western blot analysis

RAW264.7 cells were seeded on 6-well plates at a density of 5×10^6^/well and incubated overnight. Cells were pre-treated with different concentration of ICE for 30 min and stimulated by LPS for different time points. Cells were then rinsed with PBS and the total protein prepared using RIPA kit (CoWin Biosciences, Beijing, China). Western blot analysis was carried out as described previously (Zhou et al., 2021).

### Acute gastritis mouse model

The 6 week old ICR mice were purchased from SPF Biotechnology Co. Ltd (Beijing, China) and were housed in standard mouse cages at 20°C with a 12 h light/dark cycle. Food and water were freely provided. 40 ICR mice were randomly divided into five groups with eight mice in each group and were named as Normal Control (NC) group, Model Control (MC) group, Low-Dose (LD) group, High-Dose (HD) group and Positive Control (PC) group, respectively. Mice in NC group and MC group were intragastrical administered with 0.5% CMC-Na, LD and HD group were intragastrical administered with ICE at 35 and 100 mg/kg of body weight. 35 mg/kg of ranitidine were intragastrical administered as the positive control. All the mice were administered 6 times in 3 days and an 18 h starvation were carried out after the last administration. To build the acute gastritis model, all the administered mice were treated with 400 μl HCL/EtOH solution (150 mM HCL in 60% ethanol) for 1 h, then the mice were sacrificed and the stomach were taken out. Total RNA of the stomach were prepared after grounded the tissues to powder with liquid nitrogen. The mRNA expression levels of iNOS, TNFα, IL-1β and IL-6 in stomach were analyzed by RT-qPCR analysis. All animal experiments in accordance with those approved by the Institutional Animal Care and Use Committee at the Affiliated Huaian No. 1 People’s Hospital (approval number: DW-P-2022-001-01).

### Statistical analysis

Results in the present study were represented as the mean ± SD (standard deviation). The significance was analyzed between the two groups using Student’s *t*-test and *p*-values less than 5% were considered to be statistically different.

## Results

### UPLC-QTOF-MS analysis of the *I*. *compacta* ethanol extract

**FIGURE 1:**
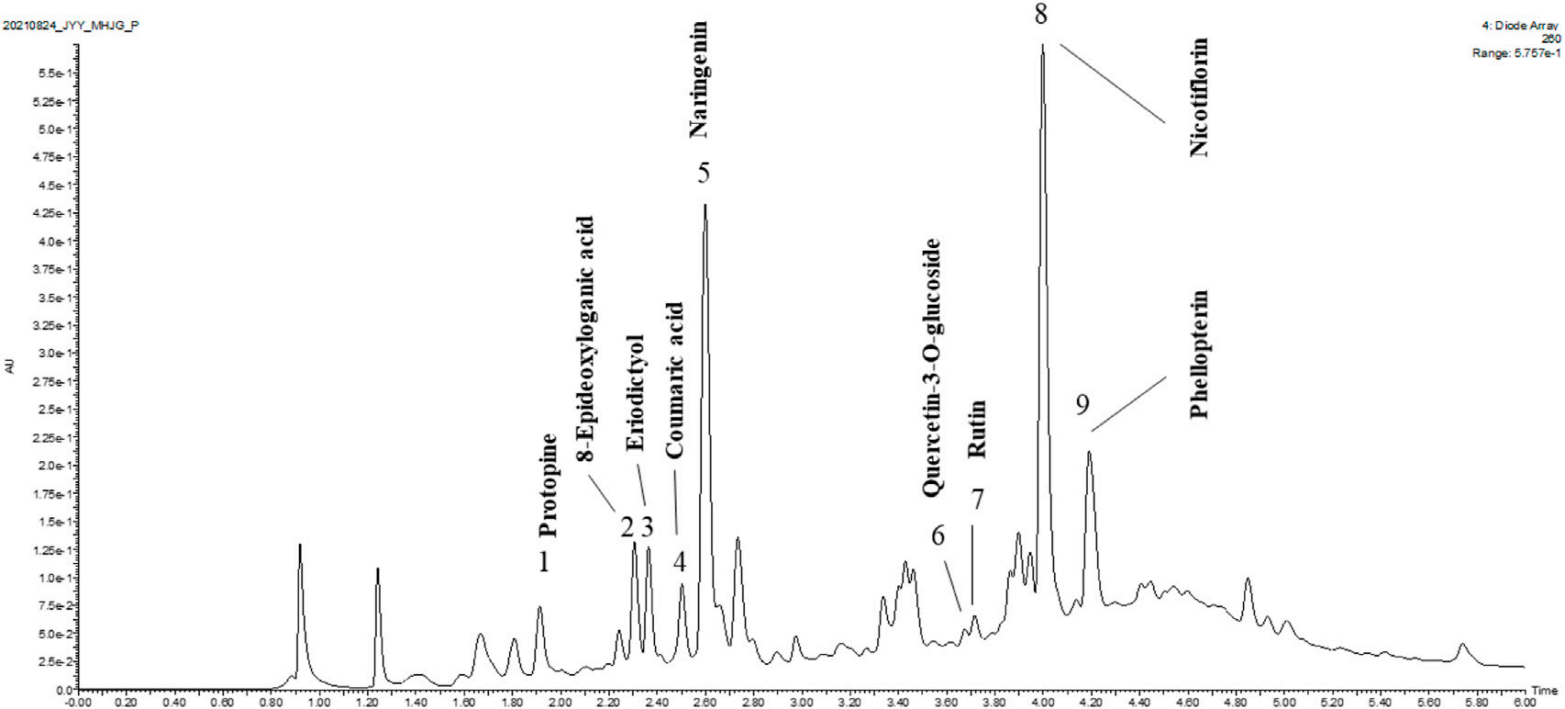
UPLC-QTOF-MS analysis of ICE.

UPLC-QTOF-MS was used to identify the major compounds in ICE. Nine chromatographic peaks were identified in the ICE profile (Figure 1). Comparing the retention times and ultraviolet and MS spectra of the samples with those of the standards (Table 2), nine main compounds were identified: peaks 1-9 were protopine, 8-epideoxyloganic acid, eriodictyol, coumaric acid, naringenin, quercetin-3-O-glucoside, rutin, nicotiflorin, and phellopterin, respectively.

**TABLE 2:** The main compounds in ICE identified based on UPLC-QTOF-MS analysis.

NO.	t_R_ (min)	Formula	Observed *m/z* [M + H]^+^ [M-H]^-^	Maximum wavelength (nm)	Error (ppm)	Fragment ions	Identification
1	1.91	C20H19NO5	353.1284	240	4.4	176.0719	Protopine
						145.0146	
2	2.30	C16H24O9	361.1501	241	2.3	163.0582	8-Epideoxyloganic acid
						132.0811	
						85.0287	
3	2.36	C15H12O6	289.0716	241	4.5		Eriodictyol
4	2.50	C9H8O3	165.0555	230	5.5	79.0546	Coumaric acid
5	2.60	C15H12O5	273.0766	250	0.91	271.0618	Naringenin
6	3.72	C21H20O12	465.1084	322	4.4	307.0716	Quercetin-3-O-glucoside
						133.0648	
7	3.72	C27H30O16	611.1640	322	4.4	463.0905	Rutin
						286.0488	
						151.0407	
8	4.00	C27H30O15	595.1683	330	4.3	315.0887	Nicotiflorin
						287.0571	
						249.0734	
9	4.19	C17H16O5	301.1074	330	1.0		Phellopterin

### Effects of ICE on RAW 264.7 cell viability

**FIGURE 2:**
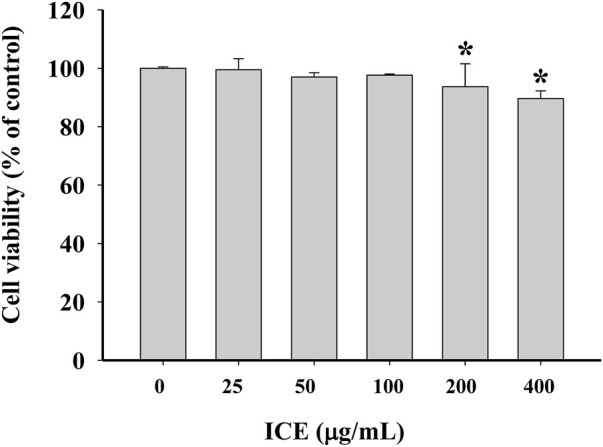
Effect of ICE on RAW264.7 cell viability after a 24 h treatment. *p < 0.05versus the control group.

The cytotoxicity of ICE on RAW264.7 cells was estimated using the MTT assay. A high ICE concentration did not significantly affect cell viability (Figure 2). RAW264.7 cell viability was decreased slightly on treatment with 200 or 400 μg/ml ICE. When the ICE concentration was 100 μg/ml or less, there was almost no effect on cell viability. Therefore, the ICE concentrations used in subsequent studies were 100 μg/ml or lower.

### Effects of ICE on NO generation and the release of proinflammatory cytokines

**FIGURE 3:**
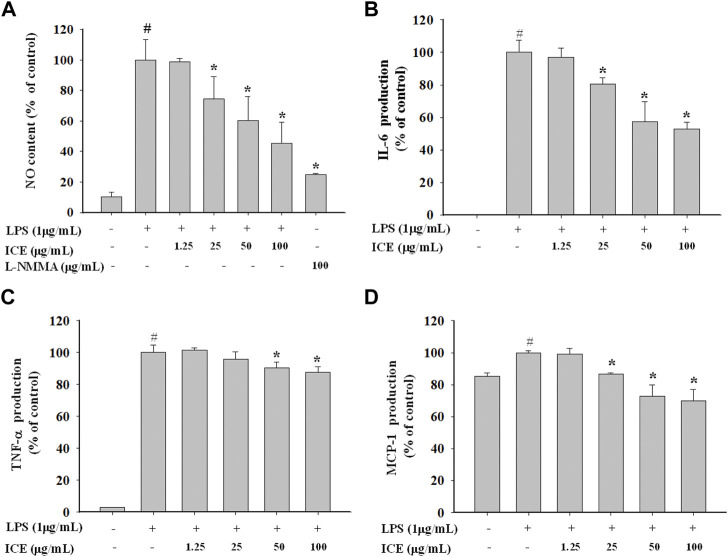
Effects of ICE on NO generation **(A)** and pro-inflammatory cytokines levels **(B–D)** in LPS-stimulated RAW 264.7 cells. The NO content and the cytokines levels were measured using the Griess reagent and Cytometric bead array (CBA) assay. *p < 0.05 versus the LPS treated cells (LPS); #*p* < 0.05 versus the normal cells (Control).

To validate the anti-inflammatory effect of ICE, the NO generated was measured (Figure 3A). The NO content in LPS-stimulated RAW 264.7 cells was increased markedly compared with controls. However, in the presence of 12.5, 25, 50, and 100 μg/ml ICE, NO production significantly reduced to 98.62%, 74.63%, 60.31%, and 45.29% of control, respectively. L-NMMA (100 μg/ml) inhibited NO production to 24.88% in LPS-stimulated RAW264.7 cells. The proinflammatory factors IL-6, MCP-1 and TNF-α were assessed with the CBA assay, and ICE reversed the increased pro-inflammatory cytokine production in a dose-dependent manner (Figures 3B–D).

### Effects of ICE on PI3K/AKT expression and phosphorylation

**FIGURE 4:**
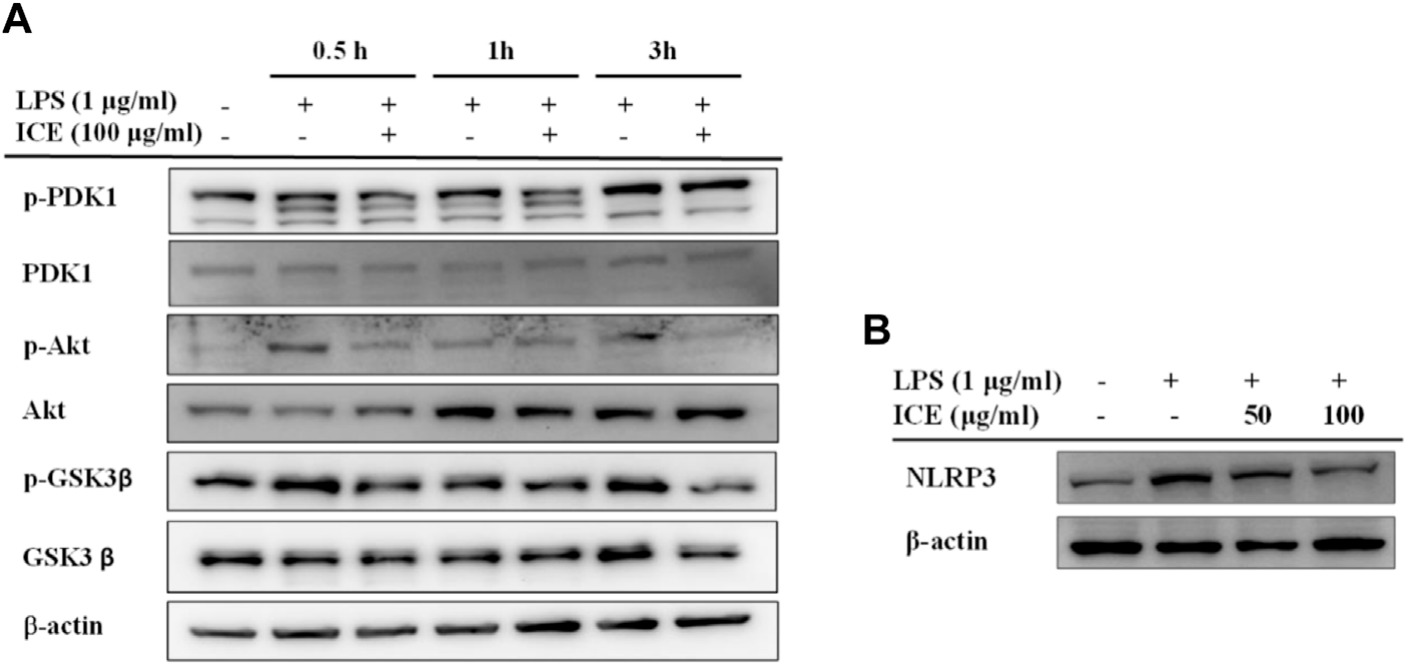
Effects of ICE on PI3K/AKT signalling pathway in LPS-stimulated RAW 264.7 cells. **(A)** The levels of total or phosphorylated PDK1, Akt and GSK-3β, and **(B)** the level of NLRP3 were identified by western blot. *β*-actin was used as a control.

To validate whether ICE exerts its anti-inflammatory activity *via* the PI3K/AKT signalling pathway, PDK1, AKT, and GSK3β proteins, and the phosphorylation thereof, as well as NLRP3 were measured in LPS-stimulated RAW 264.7 cells with or without ICE treatment by Western blotting (Figures 4A,B). This revealed that ICE downregulated PDK1, AKT and GSK3β phosphorylation (Figure 4A), meanwhile, ICE also downregulated the expression level of NLRP3 (Figure 4B). Therefore, we speculate that the PI3K/AKT signalling pathway and NLRP3 are involved as the targets of ICE anti-inflammatory activity.

### Effects of ICE on gene expression at the transcriptome scale in LPS-stimulated RAW 264.7 cells

**FIGURE 5:**
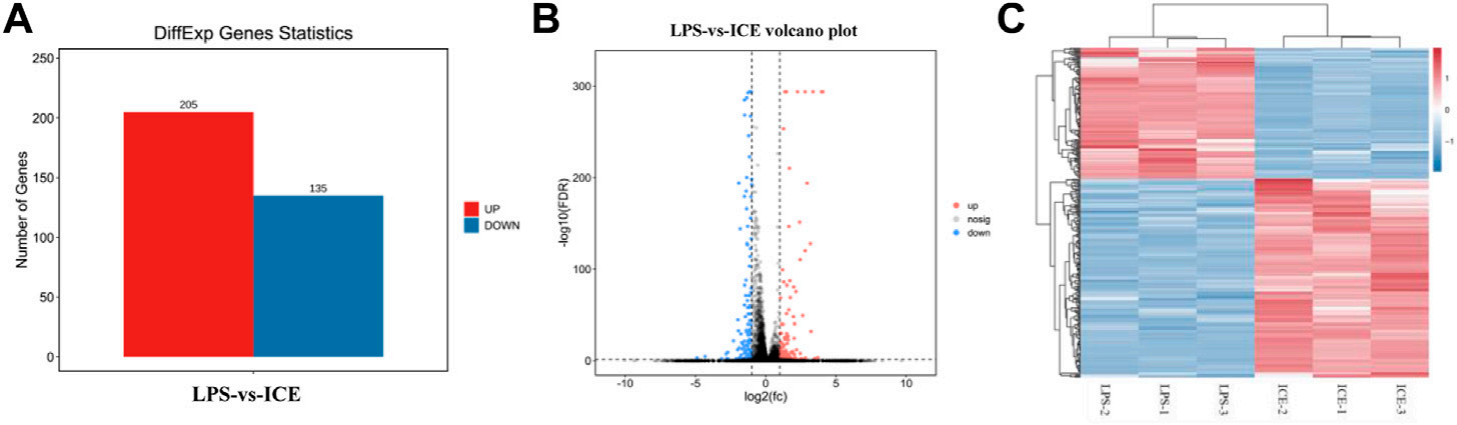
RNA-seq analysis revealed genes that response to ICE treatment in LPS-stimulated RAW 264.7 cells. DEGs between LPS-vs-ICE groups were selected and statistics analysed **(A)** and displayed in a volcano plot **(B)**; Cluster analysis of DEGs were showed in a heat map **(C)**. Red colour in the charts indicates the genes were upregulated and blue colour indicates the genes were downregulated.

To understand the mechanisms underlying ICE anti-inflammatory activity at a broader scale, the transcriptomes of LPS-stimulated and ICE-treated RAW 264.7 cells were analysed using RNA-Seq. This revealed 340 DEGs, including 205 upregulated and 135 downregulated genes (Figure 5A). The DEGs are displayed in a volcano plot (Figure 5B) and heatmap (Figure 5C). DEGs that clustered in the heatmap showed repeatable gene responses to ICE treatment among experimental replicates.

**FIGURE 6:**
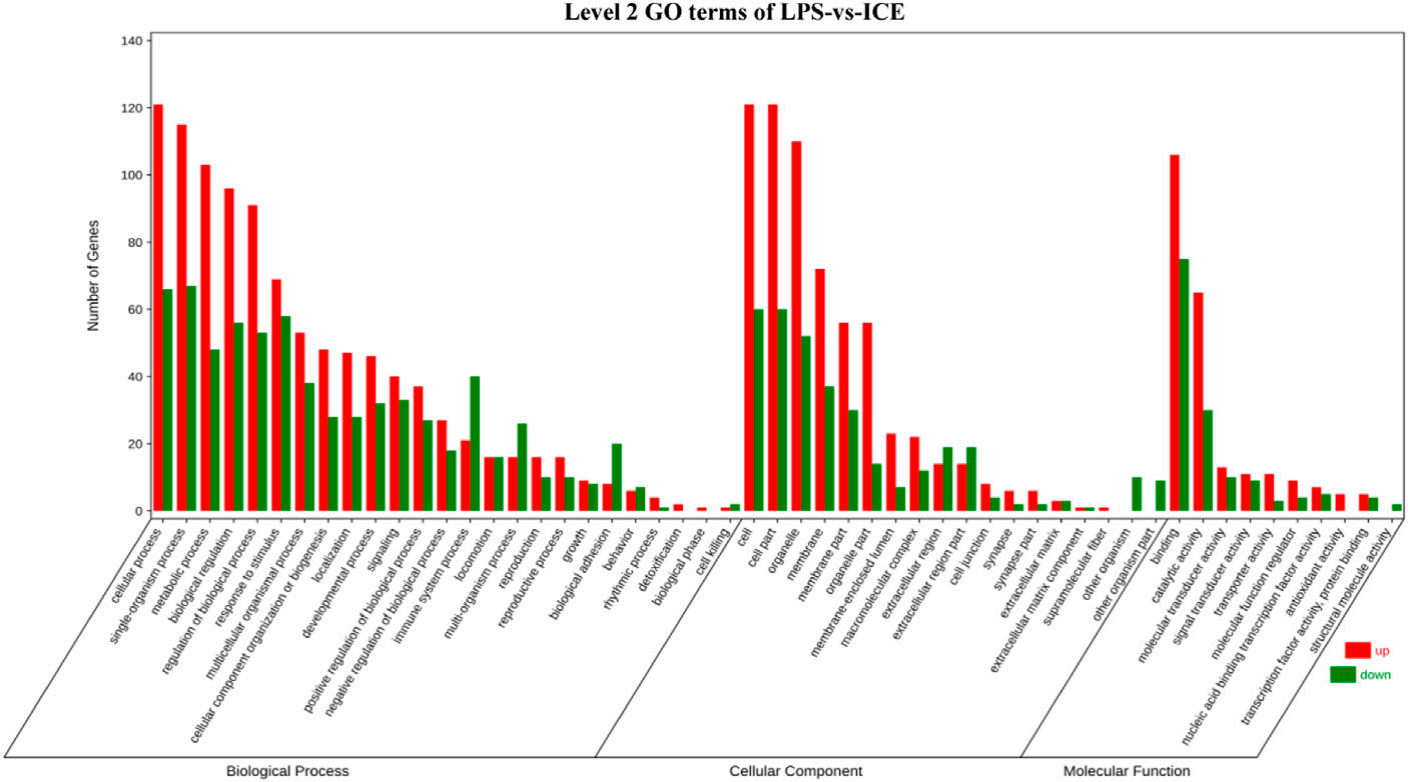
Level 2 GO terms of LPS-*vs*-ICE in the GO enrichment analysis of DEGs.

**FIGURE 7:**
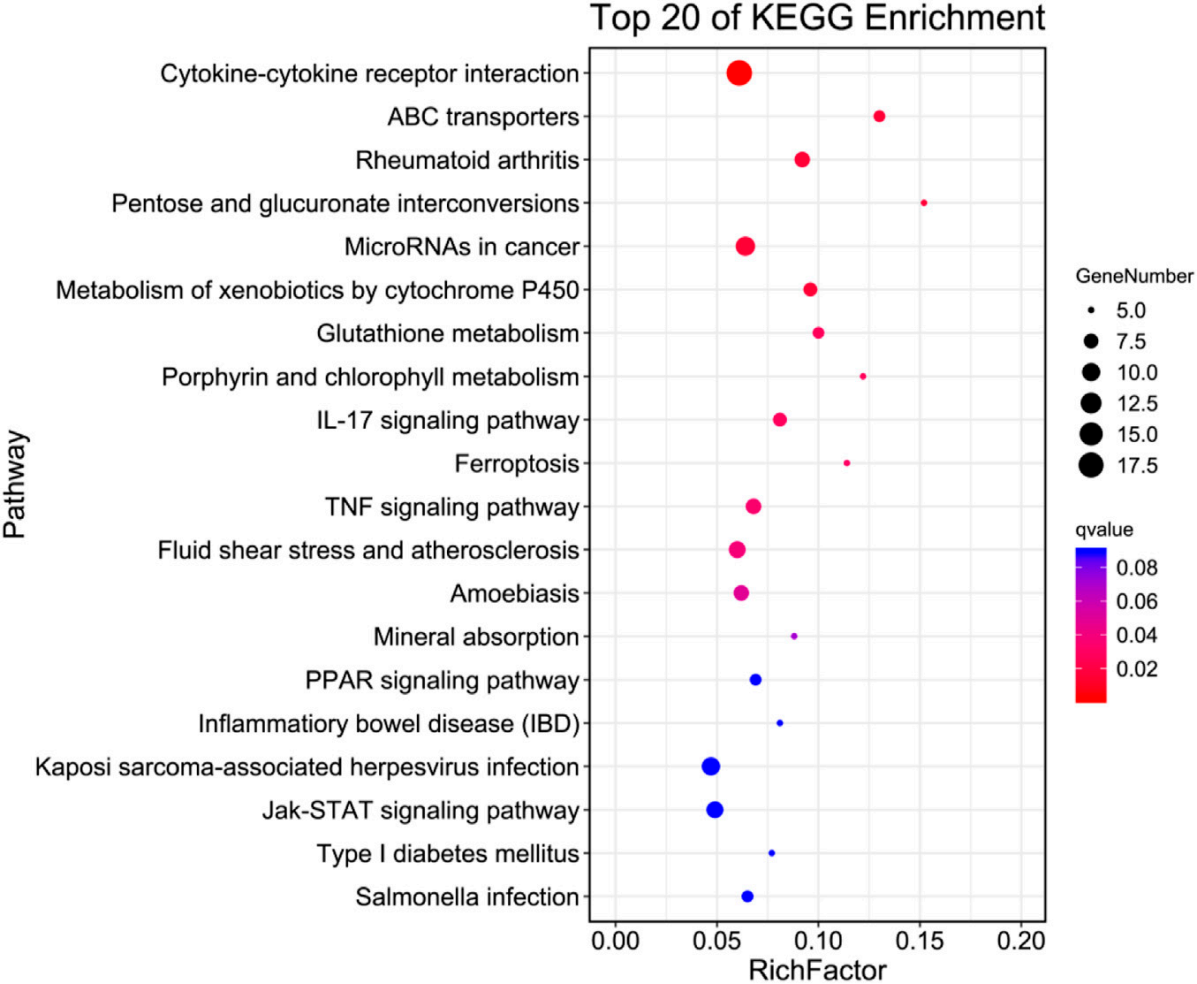
Top pathways in the KEGG enrichment analysis of DEGs.

To gain insight into the functions of selected DEGs, GO and KEGG enrichment analyses were conducted. In the GO enrichment analysis (Figure 6), DEGs were enriched in biological process (BP), cellular component (CC), and molecular function (MF). For BP, DEGs were mainly enriched in cellular process, single-organism process, metabolic process, biological regulation, regulation of BP, and response to stimulus. Note that in BP, several DEGs were enriched in signalling, positive and negative regulation of BP, and immune system process. For CC, DEGs were mainly enriched in cell, cell part, organelle, membrane, membrane part, organelle part, membrane-enclosed lumen, and macromolecular complex. For MF, DEGs were mainly enriched in binding, catalytic activity, molecular transducer activity, signal transducer activity, transporter activity, MF regulator, transcription factor activity, and protein binding. In the KEGG enrichment analysis (Figure 7), DEGs were significantly enriched in cytokine–cytokine receptor interaction, IL-17 signalling pathway, TNF signalling pathway, and inflammatory bowel disease.

**FIGURE 8:**
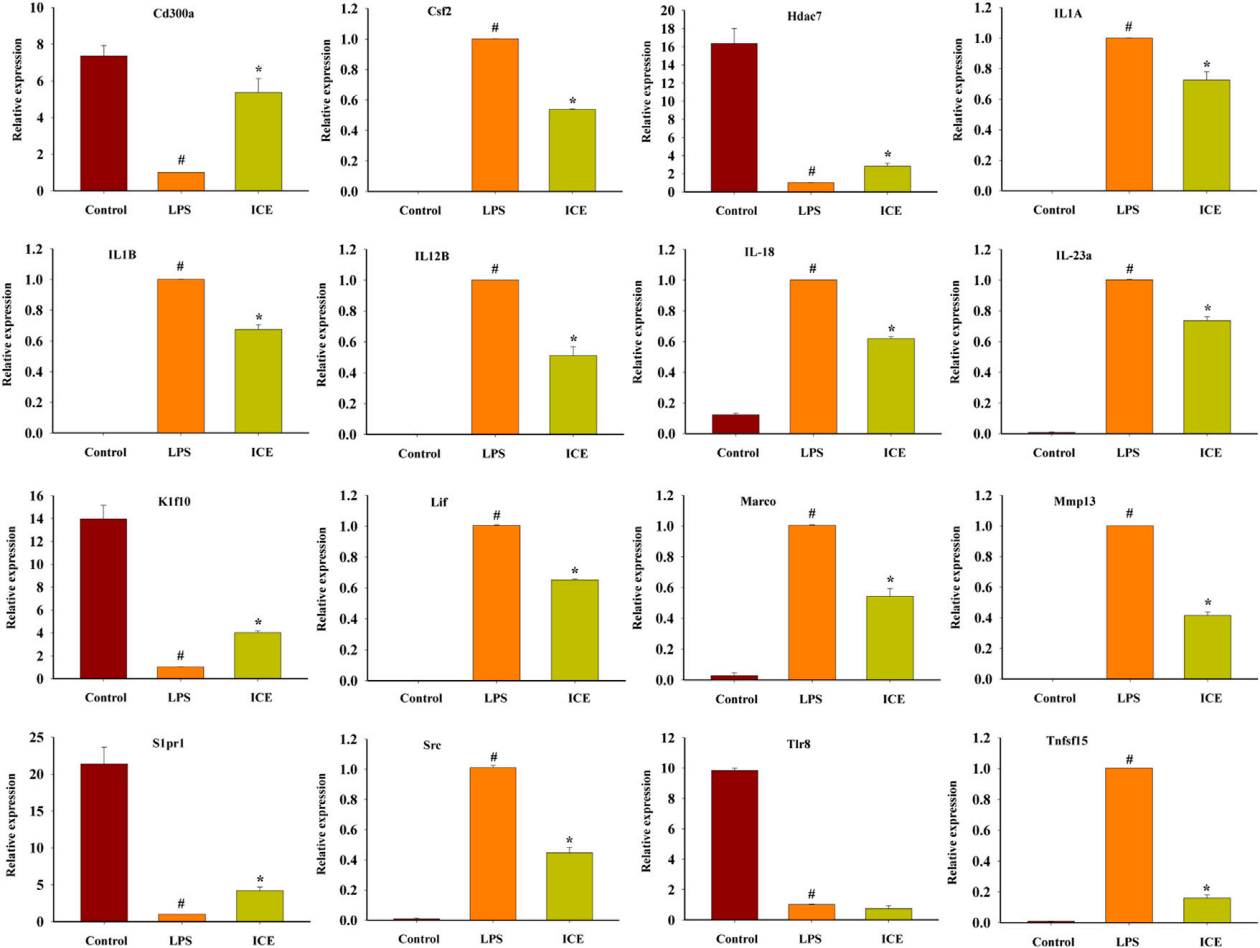
RT-qPCR analysis to validate the mRNA expression levels of selected DEGs. *p < 0.05 versus the LPS treated cells (LPS); ^#^ *p* < 0.05 versus the normal cells (Control).

To verify the reliability of the RNA-Seq data, the mRNA expression of 16 representative DEGs was assessed by RT-qPCR. The mRNA expression of these 16 DEGs showed the same trend as in the RNA-Seq analysis (Figure 8). Therefore, our RNA-Seq analysis was accurate and reliable, and reflects the expression changes of genes in response to ICE treatment.

### Anti-inflammatory effects of ICE in a mouse acute gastritis model

**FIGURE 9:**
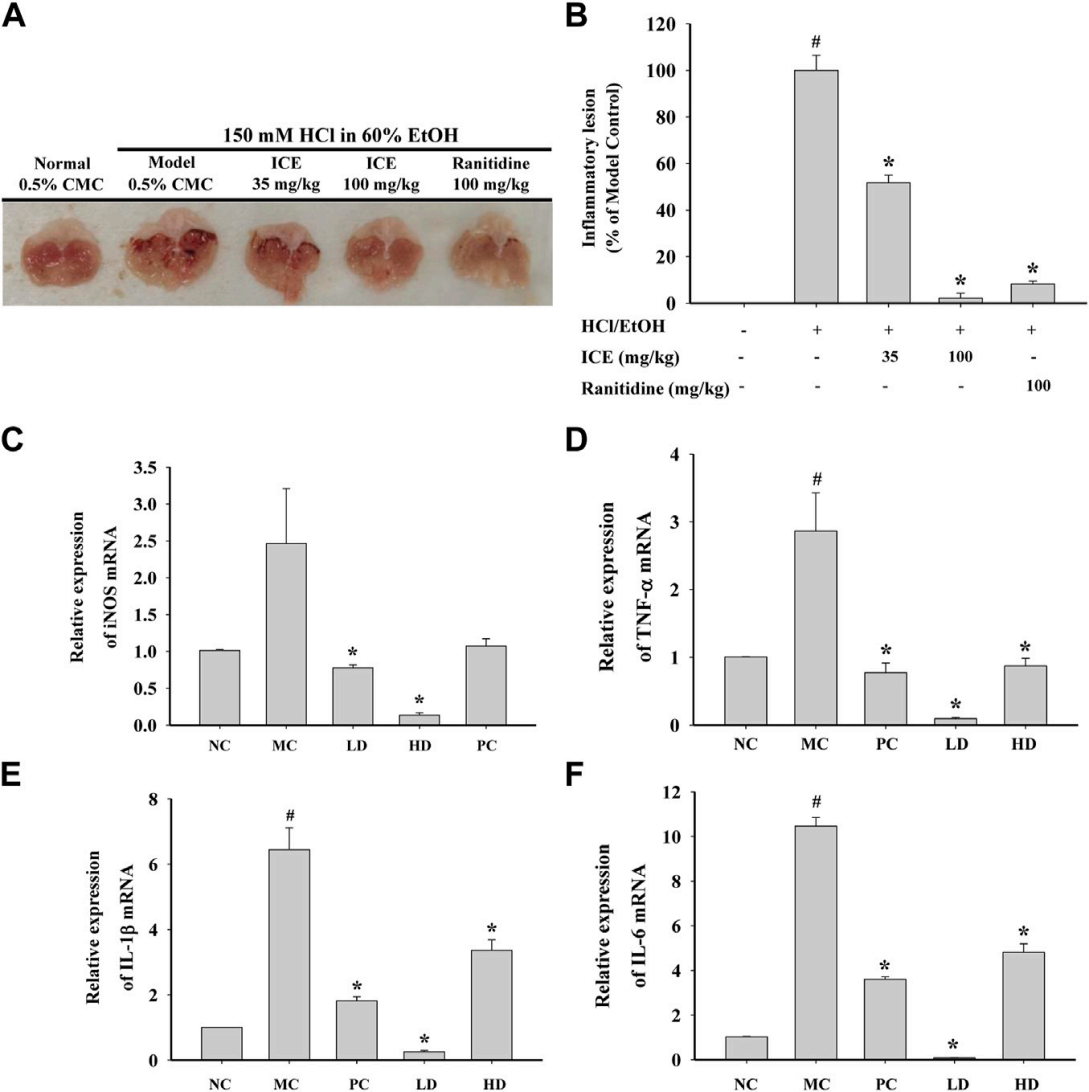
*In vivo* anti-inflammatory effects of ICE on HCL/EtOH induced gastritis model. Mice orally administered 6 times with ICE (35 mg/kg), ICE (100 mg/kg), or ranitidine (35 mg/kg) for 3 days were orally treated with HCL/EtOH for 1 h. Gstric lesions were photographed **(A)** and inflammatory lesion was quantitatively estimated by ImageJ **(B)**. mRNA expression levels of iNOS, TNF-α, IL-1β and IL-6 in the gastric tissue **(C–F)**. *p < 0.05 versus the LPS treated cells (LPS); #*p* < 0.05 versus the normal cells (Control).

To study the anti-inflammatory activity of ICE *in vivo*, a mouse HCL/EtOH-induced acute gastritis model was established, and ICE or the positive control ranitidine was administered intragastrically. Compared with the control, the gastric tissue in the MC group showed severe inflammatory lesions; the lesions were less obvious in the LD, HD, and PC groups (Figures 9A,B). The inflammatory lesions in the HD group were markedly less severe than in the LD group, indicating that ICE has dose-dependent anti-inflammatory activity. To validate the anti-inflammatory activity of ICE in the acute gastritis model at the molecular level, the mRNA expression of iNOS and the proinflammatory cytokines TNF-α, IL-1β, and IL-6 in gastric tissue in each group was detected by RT-qPCR. The iNOS, TNF-α, IL-1β, and IL-6 mRNA expression decreased significantly with ICE administration in a dose-dependent manner (Figures 9C–F), validating the *in vivo* anti-inflammatory activity of ICE.

## Discussion

As a widely used traditional Tibetan medicine, *I*. *compacta* reduces inflammation and is used to treat otitis media (The State Pharmacopoeia Commission of the People’s Republic of China, 1995). According to the Tibetan Medicine Record, *I*. *compacta* can be used as red “Ou-qu” to prevent Qi-stagnation and turgidity, and to treat otopathy and cough; it can also been used to reduce abdominal distention (Yang, 1991; Dge-bśes, 1986). In the last decade, a few papers have reported the pharmacological activity of *I*. *compacta* based on modern pharmacological techniques. A phytochemical analysis revealed that *I*. *compacta* contains several bioactive compounds, including polyphenols, flavonoids, alkaloids, and phenylethanoid glycosides (Guo et al., 2019). In this study, the compounds in ICE were analysed using UPLC-QTOF-MS, which identified nine compounds: protopine, 8-epideoxyloganic acid, eriodictyol, coumaric acid, naringenin, quercetin-3-O-glucoside, rutin, nicotiflorin, and phellopterin (Figure 1; Table 2). Several of them were reported with anti-inflammatory properties, such as protopine (Saeed et al., 1997), eriodictyol (Lee, 2011), and naringenin (Ahmed et al., 2018). These compounds may act as the phytochemical basis of ICE to exert its anti-inflammatory property.

Previously, we found that phenylethanoid glycosides extracted from *I*. *compacta* root had hepatoprotective effects in a carbon tetrachloride-induced liver HepG2 cell injury model, imparted *via* antioxidation and NF-κB downregulation (Shen et al., 2015; Wu et al., 2016). The trichloromethane fraction of *I*. *compacta* root, designated R2 in a previous report, exerted anti-proliferation effects on AGS-EBV cancer cells by regulating related protein expression and inducing EBV lytic replication, apoptosis, and G0/G1 arrest (Zhang et al., 2016). Besides antioxidant effects, an aqueous extract of *I compacta* showed dose-dependent analgesic effects in the formalin test (Guo et al., 2019).

This work investigated the anti-inflammatory activity of *I*. *compacta* in LPS-stimulated RAW 264.7 cells. Compared with controls, RAW264.7 cells treated with 1 μg/ml LPS generated large amounts of NO, while LPS-stimulated RAW264.7 cells pre-treated with different ICE concentrations generated significantly less NO, in a dose-dependent manner (Figure 1). The intercellular messenger NO has many roles in the immune system. Activated macrophages release NO in response to infection (Bogdan, 2001). As an immune system modulator, NO indicates inflammation (Tripathi et al., 2007). Therefore, the inhibitory activity of ICE on NO generation in LPS-stimulated RAW 264.7 cells suggests anti-inflammation activity. The levels of the pro-inflammatory cytokines IL-6, MCP-1, and TNF-α in LPS-stimulated RAW 264.7 cells also decreased after ICE treatment (Figure 3), verifying the anti-inflammatory activity of ICE.

PI3K/AKT is an important signalling pathway that controls many cellular processes, including cell division, autophagy, survival, and differentiation; it also modulates the inflammatory response (Fruman et al., 2017). The PI3K/AKT signalling pathway involves many anti-inflammatory compounds. The compounds that we identified in ICE, including protopine, eriodictyol, coumaric acid, naringenin, rutin, and phellopterin, regulate the PI3K/AKT signalling pathway to exert their pharmacological activities (Zhang et al., 2012; Lim and Song, 2016; Lim et al., 2017; Lim et al., 2017; Bao et al., 2018; Zhou et al., 2019; Li et al., 2020; Liu and Li, 2021; Nie et al., 2021). Hence, we speculate that the PI3K/AKT signalling pathway is involved in the mechanism underlying the anti-inflammatory activity of ICE. To test this hypothesis, the expression and phosphorylation of PDK1, AKT, and GSK3β in LPS-stimulated RAW 264.7 cells was assessed by Western blotting; the ICE pre-treated cells showed downregulated PI3K expression and p-PDK1, p-AKT, and p-GSK3β phosphorylation (Figure 4A). NLRP3 inflammasome was assembled of NLRP3, ASC and pro-caspase-1, the activation of NLRP3 inflammasome can leads to the activation of caspase-1 and further to release the proinflammatory cytokines IL-1β and IL-18. The activation of NLRP3 inflammasome were reported to relating with a wide range of diseases, such as type 2 diabetes, Alzheimer’s disease, obesity, cerebral and myocardial ischemic diseases, and a variety of auto-immune and auto-inflammatory diseases (Fusco et al., 2020). Therefore, pharmacological research on NLRP3 inhibitors gained widely concern in the resent years. In the past decades, several inhibitors targeting on NLRP3 inflammasome have been reported (Shao et al., 2015), including Oridonin, OLT1177, Tranilast and many other agents (Zahid et al., 2019). In the present study, the expression level of NLRP3 in RAW264.7 cells was revealed been downregulated by ICE treatment (Figure 4B). These results indicate that the PI3K/AKT signalling pathway and inhibition of NLRP3 inflammasome are involved in the anti-inflammatory effects of ICE.

To better understand the anti-inflammatory mechanism of ICE, RAW 264.7 cell genes that responded to ICE treatment were analysed at the transcriptome level using RNA-Seq, this revealed 340 DEGs (205 upregulated, 135 downregulated; Figure 5A) enriched in several inflammation-related GO terms and KEGG pathways (Figures 6, 7). In the GO enrichment analysis, DEGs were enriched in inflammation-related biological processes, such as cellular processes, biological regulation, BP regulation, response to stimulus, signalling, positive/negative regulation of BP, and immune system process. In the MF sub-ontology, DEGs were mainly enriched in binding, catalytic activity, molecular/signal transducers activity, MF regulator, and transcription factor activity. Therefore, ICE exerts its anti-inflammatory activity *via* these biological processes and molecular functions.

IL-17 is induced during bacterial infection, and in turn induces the production of inflammatory cytokines including IL-1, GM-CSF, TNF-α, and IL-6, and chemokines such as MCP-1, MCP-3, and MIP-3A (Qian et al., 2010). Therefore, IL-17 plays critical roles in host immunity and inflammation. IL-17 activates several inflammation-related signalling pathways, including NF-κB, MAPK, C/EBPs, PI3K, and STAT (Li X. et al., 2019). In this study, the top pathways in the KEGG enrichment analysis were the IL-17 signalling pathway and other inflammation-related pathways, including the TNF, PPAR, and Jak-STAT signalling pathways. Therefore, we speculate that ICE modulates the inflammatory response *via* the IL-17 signalling pathway and other related pathways.

Alcohol abuse can cause severe gastric mucosa inflammatory lesions (Repetto and Boveris, 2010). Therefore, to study the anti-inflammatory activity of ICE *in vivo*, a mouse ethanol-induced acute gastritis model was used to assess the protective effects of ICE against inflammatory lesions. Oral ICE strongly ameliorated gastric inflammation by downregulating pro-inflammatory expression, similar to the anti-gastritis effects of plants such as *Alisma canaliculatum*, and *Geranium koreanum* (Kim et al., 2018; Nam and Choo, 2021).

## Conclusion

This study demonstrated that ICE exerts anti-inflammatory activity. The PI3K/AKT signalling pathway may be involved in the mechanisms by which ICE exerts its activity. At the transcriptome level, ICE treatment regulated hundreds of genes, many of which are involved in inflammation-related biological processes and functions, and in signalling pathways *via* which ICE exerts its anti-inflammatory effects.

## Data Availability

The data generated for this study can be found with the accession number CNP0003554 here: [https://db.cngb.org/search/project/CNP0003554/].
